# Eleventh International Medical Congress

**Published:** 1894-01

**Authors:** A. Jacobi

**Affiliations:** 110 W. 34th Street, New York


### ELEVENTH INTERNATIONAL MEDICAL CONGRESS.

A letter directed to the undersigned by the Secretery-General of the Eleventh International Medical Congress and dated December 18th, 1893. contains the following communications:

"American members will pay on the English, French and Italian railways single fares for double journeys, and will obtain a reduction of twenty per cent. on fares for Italian round-trip tickets.

"The documents required for their identification will be sent to you in January, and Americans intending to visit the Congress will have to apply to you for them.

"Full particulars concerning the journeys will accompany the documents.

"Messrs. Thos. Cook & Son, London, Paris, Rome and Naples, should be applied to for accommodation and for tickets for the excursions at Rome, Naples and to Sicily. Such excursions will be arranged at Rome under the guidance of Mr. Forbes, member of several scientific societies and correspondent of the "Times"—for Naples, three days, including Vesuvius, Pompey, Capri, Sorrento, Castellamare, Bajae, etc—for Sicily, ten days from Naples, including Messina, Taormina, Catania, Girgenti, Siracusa, Palermo, and return to Naples.

"The fares for members of the Congress will be considerably reduced and comprise hotel accommodations, carriages, boats, etc.—about 70 frcs. each for the three days, and 285 frcs. for the ten days.

"Full particulars concerning these excursions will be contained in a leafleat to be added to the instructions and documents of the journey."

From former communications the following are herewith quoted: The member's fee is five dollars, that of their wives or adult relations two dollars each. Checks or money orders may be sent to Prof. L. Pagliani, Rome, Italy. Credentials have been promised in the near future. When they arrive (none were received last year), they may be too late for many who have started or are about to start. The undersigned, who is not informed of the causes of delay, proposes to supply in as official a form as he thinks he is justified in doing, credentials which are expected to be of some practical value. The North German Lloyd has promised to recognize them. It is suggested besides, that a passport may increase the traveler's facilities.

Only the North German Lloyd (22 Bowling Green) and the Compagnie Generale Transatlantique (3 Bowling Green) have thought fit to grant any reductions to Congressists.

The reductions on Italian railways are available from March 1st to April 30th.

A. JACOBI, M. D.

January 11th, 1894. 110 W. 34th Street, New York.

